# Supplementary material for: Contagion on complex networks with persuasion
Source: Sci Rep. 2016 Mar 31;6:23766. doi: 10.1038/srep23766 (PMC4815014; doi:10.1038/srep23766)
Supplement: Supplementary Information [file srep23766-s1.pdf]

Supplementary information for "Contagion on complex networks with persuasion"

Wei-Min Huang[1], Li-Jie Zhang[2,3], Xin-Jian Xu[1,3,a] and Xinchu Fu[1,3]

[1]*Department of Mathematics, Shanghai University, Shanghai 200444, China*

[2]*Department of Physics, Shanghai University, Shanghai 200444, China*

[3]*Institute of System Sciences, Shanghai University, Shanghai 200444, China*

(Dated: December 15, 2015)

[a] Correspondence to: xinjxu@shu.edu.cn

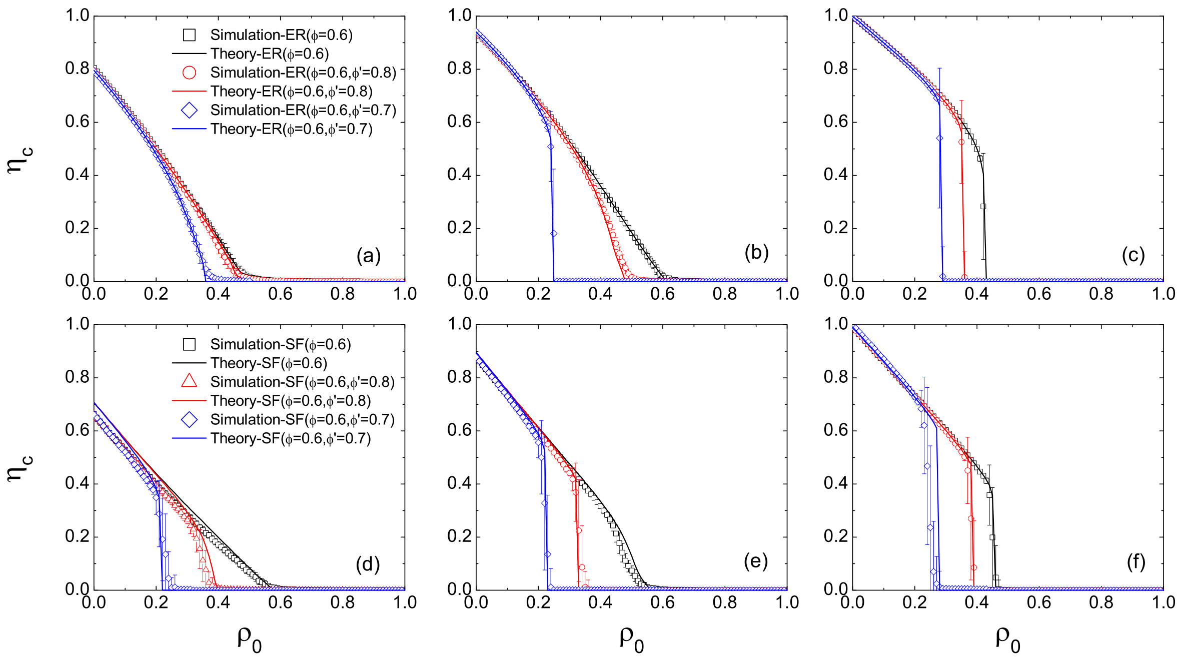

FIG. S1. Normalized size of the giant component of inactive nodes η_c in the stable states as a function of the seed fraction ρ_0. The adoption threshold is $\phi = 0.6$. Symbols are simulation results on ER (upper panel) and SF (lower panel) networks of $N = 10^4$ nodes and average degree $z = 2$ (left column), 3 (middle column) and 10 (right column), respectively. All the results are averaged over 50 realizations of the model, each of which is performed on 50 network configurations.

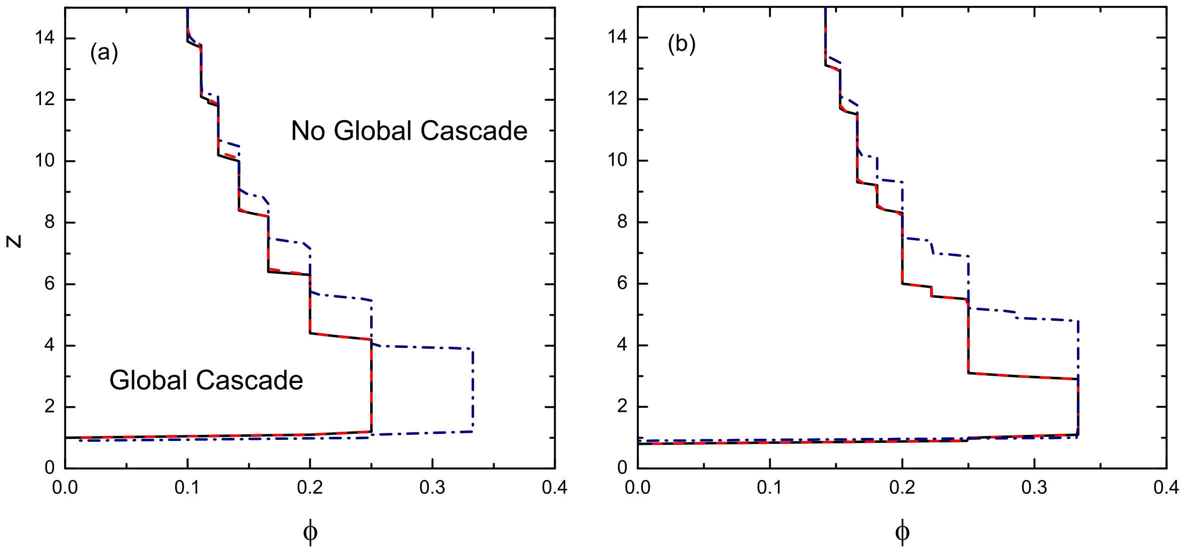

FIG. S2. The phase diagrams of ρ on the (ϕ, z) plane for ER (a) and SF (b) networks, respectively. The seed fraction is $\rho_0 = 10^{-3}$.

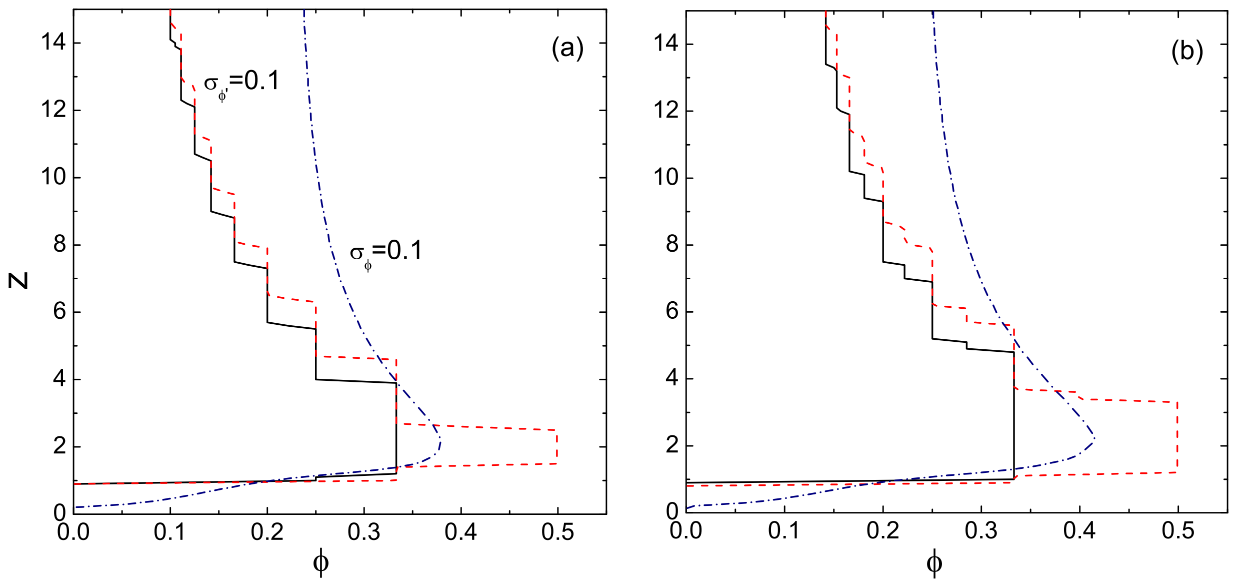

FIG. S3. Cascade windows on the (ϕ, z) plane in ER networks with seed fraction $\rho_0 = 10^{-3}$ (a) and 10^{-2} (b), respectively. Red dash and blue dot dash lines correspond to normal distributions of adoption and persuasion thresholds, respectively.
